# Supplementary material for: Respiratory Syncytial Virus Sequesters NF-κB Subunit p65 to Cytoplasmic Inclusion Bodies To Inhibit Innate Immune Signaling
Source: J Virol. 2020 Oct 27;94(22):e01380-20. doi: 10.1128/JVI.01380-20 (PMC7592213; doi:10.1128/JVI.01380-20)
Supplement: Supplemental file 1 [file JVI.01380-20-s0003.pdf]

**Supplementary figure legends.**

**Supplementary video 1: Animated projection of RSV IBs.** MDBK cells infected with bRSV; nine Z-section images, 0.9 µm apart, taken through the cell to allow quantification of N positive puncta were concatenated in the Image J software to generate this animation.

**Supplementary video 2: A 3D projection of RSV IBs.** The concatenated z-stack imaging from supplementary video 1 were used to generate a 3D projection of RSV IBs, with 1 pixel spacers added between adjacent z-stacks to achieve the required 3D effect. The projection was performed in Image J software.
